# Ultrasound-guided percutaneous antegrade pyelography for suspected ureteral obstruction in 6 pet guinea pigs (*Cavia porcellus*)

**DOI:** 10.1080/01652176.2020.1803512

**Published:** 2020-08-13

**Authors:** Dario d’Ovidio, Federica Pirrone, Thomas M. Donnelly, Adelaide Greco, Leonardo Meomartino

**Affiliations:** a Private Practitioner, Via Cristoforo Colombo 118, Arzano, Italy; b Clinica Veterinaria Malpensa, AniCura Group, Samarate, Italy; c Department of Veterinary Medicine, University of Milan, Milan, Italy; d Exotic Medicine Service, École Nationale Vétérinaire d’Alfort, Maisons-Alfort, France; e Department of Advanced Biomedical Sciences, University of Naples Federico II, Naples, Italy; f Interdepartmental Centre of Veterinary Radiology, University of Naples Federico II, Naples, Italy

**Keywords:** Guinea pig, *Cavia porcellus*, ultrasound, urography, ureteral calculi

## Abstract

**Objectives:**

To describe the feasibility and safety of ultrasound-guided percutaneous antegrade pyelography (US-PAP) in pet guinea pigs (*Cavia porcellus*) with suspected ureteral obstruction.

**Materials and methods:**

Six adult pet guinea pigs (4 females and 2 males, all intact) were evaluated for suspected ureteral obstruction. The mean weight of the guinea pigs was 0.8 ± 0.25 kg (range 0.4–1.1 kg), and mean age was 4.07 ± 1.63 years (range 2–7 years). All animals were free from comorbid diseases, had clinical signs of urologic disease and were referred based on either strong clinical suspicion of, or diagnostic imaging of ureteral obstruction. Data on signalment and clinical examination findings, response to anaesthesia and imaging findings were recorded.

**Results:**

Partial ureteral obstruction was confirmed in all guinea pigs but one, in which a complete ureteral obstruction occurred. Uroliths were in both ureters of 5 cases and in both the left renal pelvis and ureters in 1 case. All guinea pigs showed a normal appetite and regular defaecation within 2 h following the procedure. No intraoperative or immediate postoperative complications were encountered after the procedure. The only complication was contrast medium leakages in the subcapsular perinephric, retroperitoneal and, in one case, peritoneal space, which caused no overt clinical consequences afterwards. In one male patient, mobilisation of the ureteral calculus occurred and the urolith was found in the urinary bladder on the radiograph taken after contrast medium injection.

**Clinical significance:**

The US-PAP technique is a useful, safe and easy-to-perform diagnostic tool in guinea pigs with hydronephrosis and hydroureter.

## 1. Introduction

Urolithiasis is common in guinea pigs (Hawkins et al. 2009; Miwa and Sladky 2016). The calculi are composed mostly of calcium carbonate (Hawkins et al. 2009; Osborne et al. 2009) and can occur in the kidneys (Steiner et al. 1939), ureters (Gaschen et al. 1998; Stieger et al. 2003; Eshar et al. 2013), urinary bladder (Fawcett et al. 2016) and urethra (Fehr and Rappold 1997). Common clinical signs vary depending on the size and location of the calculi along the urinary tract, and include stranguria, dysuria and haematuria. Affected animals may present with urine scald, anorexia, hunched posture, weight loss, and lethargy (Hawkins and Bishop 2012; Miwa and Sladky 2016).

A definitive diagnosis of urolithiasis can be achieved through several diagnostic imaging modalities that include survey radiographs, ultrasonography (US), excretory intravenous pyelography (IVP) and computed tomography (Hawkins and Bishop 2012). In dogs and cats, abdominal US and radiography are the most sensitive diagnostic tools for documenting ureteral obstruction (Berent 2011). However, multiple calculi and/or significant amounts of gas in the gastrointestinal tract may represent a limitation to using such traditional imaging modalities in guinea pigs (Hawkins and Bishop 2012). Neither radiography or US allows for determination of whether the ureteral obstruction is partial or complete. Excretory IVP is a useful tool to elucidate functional abnormalities in the kidneys or ureters, but peripheral intravenous catheters can be challenging to place because of the small size and fragility of the veins, particularly in severely dehydrated guinea pigs (Hawkins and Bishop 2012; Quesenberry et al. 2012). In addition, due to the decreased glomerular filtration rate associated with ureteral obstruction, IVP is not considered reliable to confirm ureteral obstruction or detect the sites of urine leakage in dogs (Ghali et al. 1999; Berent 2011). Finally, intravenous contrast material is a nephrotoxic risk factor in patients whose renal function is already compromised (Berent 2011).

Medical treatment is often ineffective, and surgical removal of the uroliths (through ureterotomy) is considered the treatment of choice, especially if a complete ureteral obstruction is present (Berent 2011; Bennet 2012; Quesenberry et al. 2012).

The literature on humans (see Berent 2011 for review on ureteral interventions in people) supports the use of minimally invasive endourological diagnostic and therapeutic techniques for ureteral obstructions (e.g. antegrade percutaneous nephroureterolithotomy, ureteral stenting and percutaneous nephrostomy tube placement). A systematic literature review found interventional diagnostic and therapeutic procedures are focused primarily on dogs and cats, especially since 2010, and reports on exotic pets are scarce (Berent 2011). Percutaneous renal puncture, for instance, may be quickly performed using US guidance and its use for both diagnosis and therapy is reported in humans and animals (Pfister and Newhouse 1979; Specchi et al. 2012; Etedali et al. 2019) including in a pet guinea pig (Eshar et al. 2013). Ultrasound-guided percutaneous antegrade pyelography (US-PAP) is a useful option in canine and feline patients that allows the clinician to visualise the renal pelvis and ureters, to localise ureteral obstructions, and to determine whether a partial or complete obstruction is present (Rivers et al. 1997; Adin et al. 2003; Meomartino et al. 2014; Etedali et al. 2019).

The goal of this prospective clinical study was to describe the clinical application of US-PAP in the diagnosis and evaluation for surgery of 6 guinea pigs with suspected ureteral obstruction.

## 2. Materials and methods

### 2.1. Animals

Six adult pet guinea pigs (4 females and 2 males, all intact) were evaluated for suspected ureteral obstruction. All animals had clinical signs of a urinary disorder. Four animals had a referring veterinarian (RV) diagnosis of ureterolithiasis based on radiographs or US, and two animals had no RV diagnostic imaging. All animals were free from comorbid diseases but were on prophylactic antibiotics prescribed by the RV. The mean weight of the guinea pigs was 0.8 ± 0.25 kg (range 0.4–1.1 kg), and mean age was 4.07 ± 1.63 years (range 2–7 years).

Recruitment occurred at two veterinary clinics in the Campania region, Italy. All diagnostic procedures were performed at the Interdepartmental Centre of Veterinary Radiology, University of Naples, Federico II. The study was performed under the approval of the Ethical Animal Care and Use Committee Board of the University of Naples Federico II, Department of Veterinary Medicine and Animal Production, (Protocol number: 88861-2019), and signed, informed consent was obtained from the owners. The main demographic and clinical characteristics of these 6 animals are reported in Table 1.

**Table 1.** Main demographic and clinical characteristics of 6 guinea pigs diagnosed with urolithiasis.

Animals	Sex	Age (y)	BW (kg)	Relevant history	Clinical signs	Physical examination findings	Laboratory test results		Imaging findings
							Urinalysis	Bacterial culture	
GP 1	IM	4	1.1	D	H	PA	N/A	Neg	UC
GP 2	IF	2	0.4	L, D	H	PA	H, P	Neg	UC
GP 3	IF	3.5	0.75	L, D	S/WL	PA, L	N/A	Neg	RC/UC
GP 4	IF	3.7	0.9	L, V	S	PA, L	H, P	Neg	UC
GP 5	IM	4.2	1.0	D	S/H	PA	H	Neg	UC
GP 6	IF	7	0.65	L	S/H	PA, L	N/A	Neg	UC

Animals: GP = guinea pig; Sex: IF = intact female, IM = intact male; Age: y = years; BW = body weight.

Pertinent history: D = dysorexia, L = lethargy, V = vocalisation.

Clinical signs: H = haematuria (suspected), S = stranguria, WL = weight loss.

Physical examination findings: L = lethargy, PA = painful abdomen.

Laboratory test results: H = haematuria (confirmed), P = proteinuria, N/A = not available; Neg = culture negative.

Imaging findings: UC = ureteral calculi; RC = renal calculi.

### 2.2. Imaging technique

**Figure 1.**
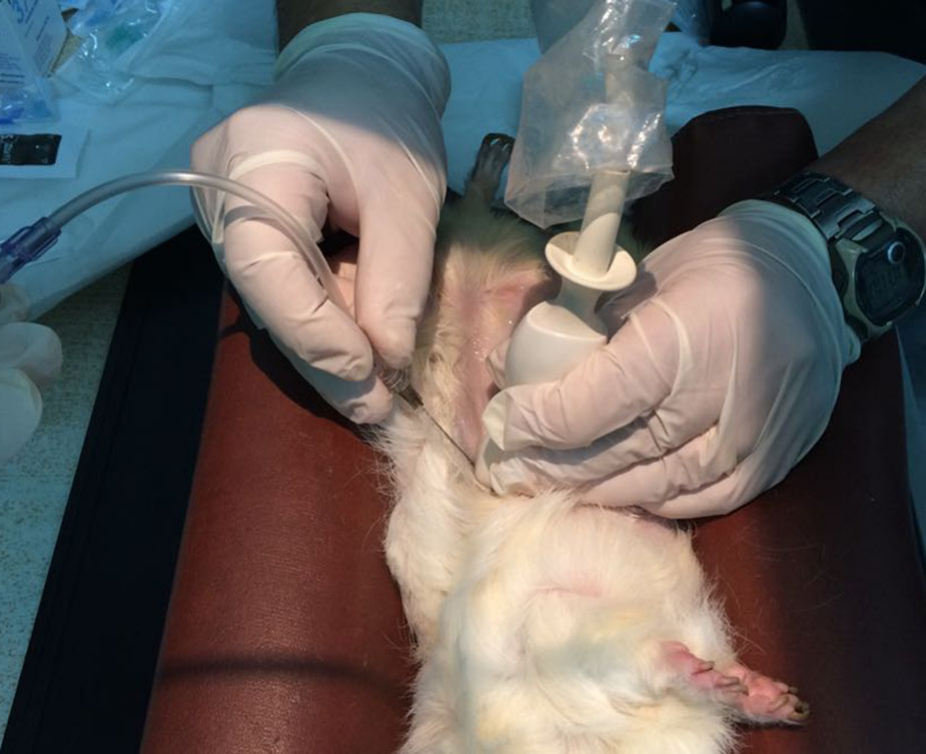
Photograph of a guinea pig undergoing ultrasound-guided percutaneous antegrade pyelography (US-PAP). The image shows the insertion of the needle under ultrasound guidance. The needle is inserted parallel to the long axis of the transducer (‘in-plane’ technique), at an angle of about 45° from the skin towards the renal pelvis. The guinea pig is supported by positioning sandbags.

**Figure 2.**
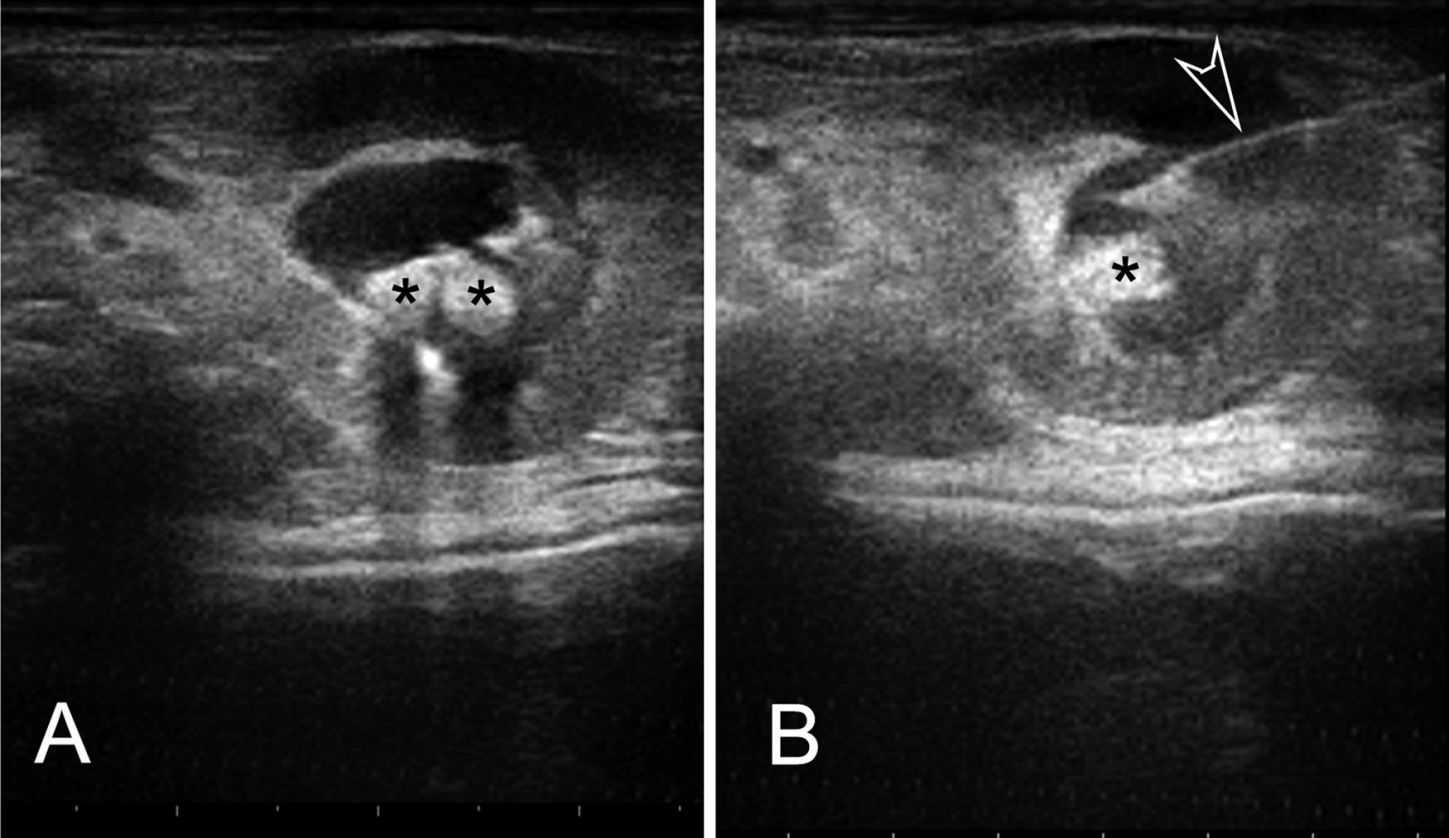
Ultrasonographic image of a guinea pig undergoing ultrasound-guided percutaneous antegrade pyelography (US-PAP). It shows a transverse scan of the left kidney immediately before the administration of contrast medium (A) and after the insertion of the needle in the renal pelvis (B). Legend: asterisks = calculi; empty arrowhead = tip of the needle in the renal pelvis.

All animals were anaesthetised with intramuscular alfaxalone (Alfaxan 1%; Jurox, UK) (5 mg/kg BW), divided in multiple sites of administration (maximum of 0.05 mL/kg BW per site) (Turner et al. 2011; Suckow et al. 2012; d’Ovidio et al. 2018) and oxygen delivery was performed through a tightly fitting face mask. The ventral abdomen, from sternum to inguinal area, was clipped and aseptically prepared for surgery in a standard fashion. Before performing the contrast study, right lateral and ventrodorsal radiographs of the abdomen were obtained. The patients were maintained in dorsal recumbency using positioning sandbags and tilted slightly on one side to better assess the kidney (Figure 1). A preliminary examination was performed with a general-purpose ultrasonographic device (MyLab Class C^©^, Esaote, Firenze, Italy) equipped with a high-frequency linear probe (13 MHz) to confirm hydronephrosis and/or ureteral calculi, and to estimate the volume of the dilated pelvis and ureter. To perform the US-PAP, the kidney and the renal pelvis were approached using a standard 8.5–10 MHz micro-convex probe, (MyLab Class C^©^, Esaote, Firenze, Italy), which has a footprint smaller than the 13 MHz probe. Under ultrasonographic guidance, a 22-gauge × 40 mm spinal needle was inserted into the renal pelvis, through the renal parenchyma, avoiding the interseptal or ileal vessels (Figures 1 and 2). After removing the stylet and attaching a three-way stopcock and extension-set, ultrasound-guided pyelocentesis was performed. A 5-mL syringe was attached, and the urine was aspirated by applying gentle negative pressure. The urine was placed in a sterile tube and saved for bacterial culture and urinalysis. Iopamidol (Iopamiro, 370 mg/mL, Bracco Imaging, Italy) diluted to 50% concentration with a 0.9% saline solution, was manually injected, ever under the US control, and a ventrodorsal radiograph was taken. The volume of iopamidol injected was half of the urine volume aspirated.

**Figure 3.**
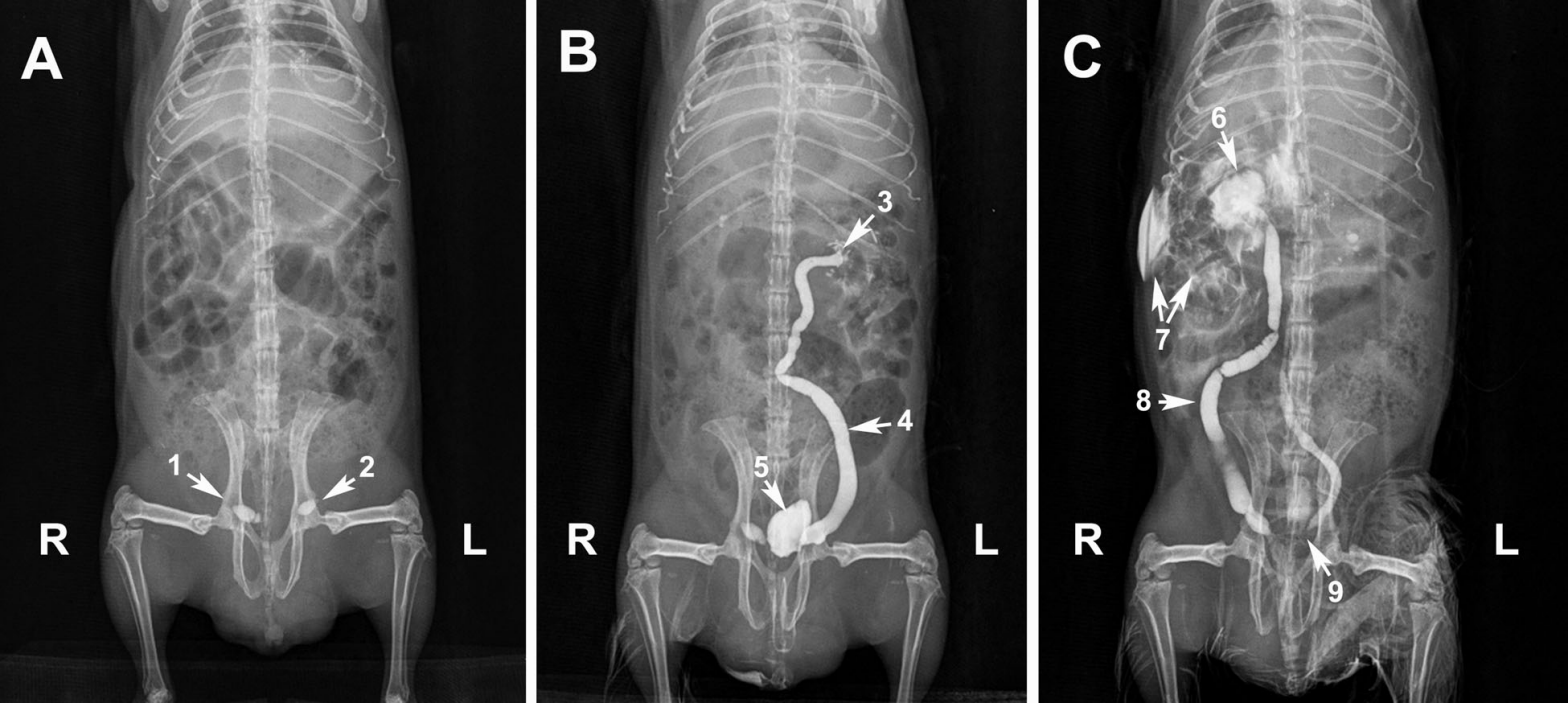
Radiographic images of a guinea pig undergoing ultrasound-guided percutaneous antegrade pyelography (US-PAP). (A) Ventrodorsal radiograph of a guinea pig in which three calculi (right side *n* = 2, left side *n* = 1) are visible in the caudal ureteral end (arrows). (B) Left percutaneous antegrade pyelography demonstrating dilation of the ureter. (C) Right percutaneous antegrade pyelography demonstrating dilation of the renal pelvis and the ureter. Leakage of the contrast medium in the peritoneal space is also visible. The left ureteral calculus is no longer visible. Legend: R = right; L = left; 1 = calculi at the caudal end of the right ureter; 2 = calculus at the end of the left ureter; 3 = left renal pelvis; 4 = left ureter; 5 = urinary bladder; 6 = right renal pelvis; 7 = contrast medium leakage; 8 = right ureter; 9 = caudal end of left ureter: the calculus has moved.

**Figure 4.**
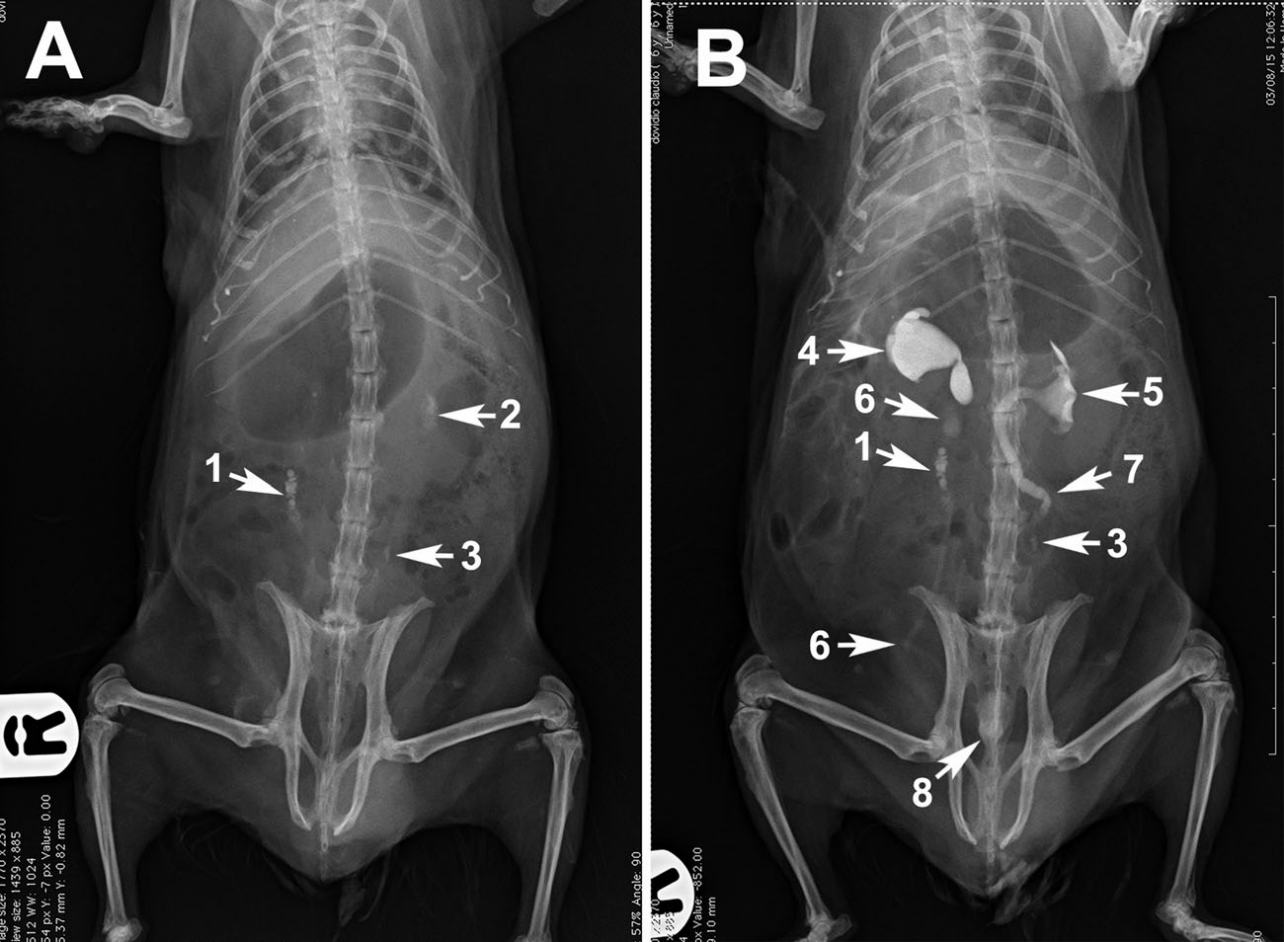
Radiographic image of a guinea pig undergoing ultrasound-guided percutaneous antegrade pyelography (US-PAP) (compare with Figure 2). (A) Ventrodorsal radiograph in which many small calculi are visible in the right and left ureters and in the left renal pelvis (arrows). (B) Percutaneous antegrade pyelography demonstrating dilation of both renal pelvises and ureters. A small amount of contrast medium is identifiable in the right ureter before and after the calculi, demonstrating a partial ureteral obstruction, while no contrast medium is visible in the left ureter after the calculi, demonstrating a complete obstruction. Legend: R = right; 1 = calculi at the right ureter; 2 = calculi at the left renal pelvis; 3 = calculi at the left ureter; 4 = right renal pelvis; 5 = left renal pelvis; 6 = cranial and caudal right ureter; 7 = cranial left ureter; 8 = urinary bladder.

If adequate filling of the renal pelvis and ureter was reached, the needle was removed. If the visualisation of the pelvis and the ureter was not complete, more contrast medium was administered (comprising an additional 30% of the urine volume aspirated) and the ventrodorsal radiograph was repeated. If contrast medium leakage was visible in a subcapsular perinephric location or the retroperitoneal space, the needle tip position was visualised using US and repositioned. Immediately after removing the needle, ventrodorsal and lateral radiographs were obtained. The same sequence was used for the contralateral pelvis and ureter (Figures 3 and 4). Postoperative care included antibiotics (trimethoprim/sulfa at 15 mg/kg BW PO q12h), anti-inflammatories (meloxicam at 0.5 mg/kg BW q24h) and US monitoring for internal haemorrhage, uroabdomen/uroperitoneum every 30 min in the first 2 h and 24 h after the procedure. A rescue analgesic regimen, consisting of butorphanol (2 mg/kg BW subcutaneous every 12 h), was planned if pain related behaviours (e.g. decreased or no appetite, immobility or reluctance to move, etc.) were observed.

## 3. Results

In all subjects, both renal pelvises and ureters were studied. Bacterial culture tests were negative (yielded no bacteria) in all cases (Table 1). Results of urinalyses are reported in Table 1. Uroliths were in the caudal ureters in 5 out of 6 cases, and in both the left renal pelvis and ureters in one case. Partial obstruction of both ureters was seen in 5 guinea pigs, and complete obstruction of the left ureter and partial obstruction of the right ureter was seen in 1 guinea pig (Figure 4(B)). The entire procedure (from the initial survey radiographs to the end of the study involving both kidneys) took a median time of 18 min (range 16–20 min). In 4 kidneys, the contrast medium leaked into a subcapsular perinephric location (*n* = 3) and/or the retroperitoneal space (*n* = 1). In the latter case, the contrast medium was also visible peritoneally (Figure 3). The median duration of anaesthesia was 27.3 min (range 25.4–31.2 min) and all guinea pigs recovered uneventfully. Median renal pelvis dilatation was 3.0 ± 0.70 mm (min 2.0 max 4.0 mm). All guinea pigs showed normal appetite and regular defaecation within 2 h following the procedure. No intraoperative or immediate postoperative complications were encountered after the procedure. In one case (GP5), the injection of contrast medium induced mobilisation of the ureteral calculus, and it was found in the urinary bladder on the radiograph taken immediately after. Bilateral ureterotomy to remove calculi was performed on one guinea pig with complete obstruction of the left ureter and partial obstruction of the right ureter (Figure 4(B)). At the 4-month follow-up, there was no recurrence of calculi, renal values were within the normal range and the patient was doing well. Of the other 5 guinea pigs, one owner elected euthanasia, and 4 owners elected not to perform surgery. Tramadol 4–5 mg/kg BW PO q12h was prescribed to the latter guinea pigs until the owners decided to proceed with either surgery or euthanasia. In addition, increase of water intake and reduction of dietary calcium (e.g. avoiding alfalfa-based diets) was also recommended. At the 1-month follow-up, calculi remained in the original position in all 4 guinea pigs that did not undergo surgery. According to the owners, the animals continued to exhibit intermittently the same clinical signs reported at the initial presentation. Mean survival time was 12 weeks.

## 4. Discussion

US-PAP is feasible and easy-to-perform in guinea pigs. Assessment of renal pelvis and ureters was possible in all animals. In agreement with previous reports, uroliths were in the caudal ureters in most cases (5 out of 6 cases) (Capello 2011; Bennet 2012). In one case, uroliths were in the left renal pelvis and in the median tract of both ureters and US was helpful to differentiate the renal calculi from renal mineralisation (Figure 4). Partial (*n* = 5) or complete (*n* = 1) ureteral obstruction occurred in all guinea pigs causing varying degrees of renal pelvis and ureter dilation (Figure 3(B)).

The most common complications associated with US-PAP reported in humans and animal patients include haematuria, temporary obstruction due to blood clots, subcapsular or perinephric haemorrhage, infection, local pain, and perforation of adjacent non-renal structures, uroabdomen or uroretroperitoneum (Rivers et al. 1997; Specchi et al. 2012; Etedali et al. 2019). There was no evidence of such complications after the percutaneous renal puncture in all the guinea pigs enrolled. Another complication described with antegrade pyelography in dogs and cats include subcapsular perinephric, retroperitoneal or peritoneal leakage of the contrast medium (Adin et al. 2003). In a study of 11 cats, leakage of contrast medium developed in 8 of 18 kidneys during antegrade pyelography and prevented diagnostic interpretation in 5 of 18 studies (Adin et al. 2003). In our sample, the contrast medium leakage occurred in 33.3% of the cases (2 of 6 animals). But this complication did not prevent diagnostic interpretation.

As animals with ureteral obstruction may have decreased renal function, contrast-induced nephropathy has been considered as a potential complication. In the feline study by Adin et al. (2003) contrast-induced nephropathy did not occur in any patient thus confirming the safety of this diagnostic procedure. In the present study, no information was available on the renal function tests of the enrolled animals, and further research is warranted to assess the consequences of US-PAP on renal function in guinea pigs. In addition, the authors highlight the importance of such information to plan the most appropriate postoperative treatment (e.g. non-steroidal anti-inflammatories *vs* opioids depending on renal function).

Another limitation we found with US-PAP was the difficulty in introducing the needle into the renal pelvis when renal pelvic dilation was minimal (2 mm). This finding agrees with previous reports in dogs and cats indicating that some renal pelvis distension (at least 5 mm) must be present to perform the procedure (Seiler 2018). The size of the kidneys (and the renal pelvis) in such small-sized patients represents a challenge even to the most skilled operators. In the present cases, the pelvis dilatation (ranging between 2 and 4 mm) made injection of the contrast medium relatively easy in animals as small as 400 grams. Although the majority of the animals enrolled presented significant amounts of gas in the gastrointestinal tract, this never interfered with the procedure, due to the position of the kidney as it was attached to the dorsal abdominal wall, and its kept immobility by aid of the US probe (Figure 1).

Several bacterial species such as *E. coli*, *Streptococcus* spp., *Staphylococcus* spp., *Corynebacterium renale* and *Proteus mirabilis* can be isolated in guinea pigs affected with urolithiasis (Hawkins and Bishop 2012). However, bacterial culture yielded no growth in all cases enrolled in this study. A possible explanation for this finding could be the ‘prophylactic/preventative’ use of systemic antimicrobials in all guinea pigs prescribed by the RVs (enrofloxacin at 5 mg/kg BW q12h), even before a final diagnosis was achieved. Such prescribing practices support the need for the prudent use of antimicrobials in companion animals.

Medical treatment is not rewarding in cases of ureteral obstruction in guinea pigs (Bennet 2012). Ureterotomy is considered the treatment of choice to remove the uroliths and relieve the obstruction and can be accomplished through a standard ventral midline celiotomy (Capello 2011). This was done successfully in one guinea pig and at the 4-month follow-up, the patient was doing well. The stones should be localised before incising to avoid the damage associated with an extensive dissection (Capello 2011). Careful manipulation of the tissues and fat surrounding the ureters should be done to avoid damages to the vascular supply (Bennet 2012). Ureteral stenting may be required to minimise the risk of suturing the ureter closed or development of strictures (Bennet 2012). However, as recurrence of urolithiasis is common in guinea pigs, the risks, benefits, and impact of the surgical treatment should be discussed with the owners. Dietary modification such as promoting the consumption of low calcium containing grass hays (e.g. timothy, oat, grass) and a wide variety of vegetables and fruits, as well as lowering the amount of pelleted food should be adopted to decrease the risk of urolith development (Hawkins and Bishop 2012).

Eshar et al. (2013) described the use of ultrasound-guided percutaneous antegrade hydropropulsion to relieve ureteral obstruction in a pet guinea pig. Using the same technique as ours, the authors injected sterile saline to dislodge the ureteral stone into the bladder. Although antegrade hydropropulsion did not completely resolve the obstruction, it allowed for clinical resolution of the associated clinical signs, as the animal did not require assisted feeding and showed no evidence of haematuria the day after the procedure (Eshar et al. 2013). Similarly, in one case in our study, injection of contrast medium dislodged a ureteral calculus into the urinary bladder, thus confirming that increased pressure in the ureter can help mobilisation and progression of the calculi through the urinary tract. Nevertheless, ureterotomy, ureteral stenting, or subcutaneous ureteral bypass placement should still be considered the treatment of choice to relieve ureteral obstruction, as in dogs and cats, regardless of their perioperative mortality rates, until further studies on the safety of the ultrasound-guided percutaneous antegrade hydropropulsion are performed.

While US-PAP seems to be a promising technique for all its described advantages, it remains an invasive method that can only be performed under general anaesthesia. If an immediate surgical intervention should be unavoidable afterwards (e.g. if total obstruction of the urinary tract occurs), this would cause an increased length of anaesthesia, which would be considered high risk because of the duration and possible impairment of kidney function.

## 5. Conclusion

Although the findings of this study are based on a small number of guinea pigs, US-PAP was as feasible and safe as in cats and dogs. This imaging modality might be applied in clinical settings as an alternative to IVP, to better evaluate ureteral calculi location and to differentiate more effectively partial or complete ureteral obstruction even when intravenous catheterisation is not possible, or when contrast medium intravenous administration is not recommended. Further studies should compare the US-PAP procedure with other diagnostic imaging techniques for evaluating the presence of complete or partial obstruction, and to assess the potential complications in azotaemic guinea pigs.
